# Networks in Coronary Heart Disease Genetics As a Step towards Systems Epidemiology

**DOI:** 10.1371/journal.pone.0125876

**Published:** 2015-05-07

**Authors:** Fotios Drenos, Enzo Grossi, Massimo Buscema, Steve E. Humphries

**Affiliations:** 1 Centre for Cardiovascular Genetics, Institute of Cardiovascular Science, University College London, London, United Kingdom; 2 MRC Integrative Epidemiology Unit, School of Social and Community Medicine, University of Bristol, Bristol, United Kingdom; 3 Medical Department—Bracco Pharmaceuticals, San Donato Milanese, Italy; 4 current affiliation: Villa Santa Maria Institute, Tavernerio, Italy; 5 Semeion Research Center of Sciences of Communication, Rome, Italy; 6 Dept. of Mathematical and Statistical Sciences, University of Colorado at Denver, Denver, CO, United States of America; Nankai University, CHINA

## Abstract

We present the use of innovative machine learning techniques in the understanding of Coronary Heart Disease (CHD) through intermediate traits, as an example of the use of this class of methods as a first step towards a systems epidemiology approach of complex diseases genetics. Using a sample of 252 middle-aged men, of which 102 had a CHD event in 10 years follow-up, we applied machine learning algorithms for the selection of CHD intermediate phenotypes, established markers, risk factors, and their previously associated genetic polymorphisms, and constructed a map of relationships between the selected variables. Of the 52 variables considered, 42 were retained after selection of the most informative variables for CHD. The constructed map suggests that most selected variables were related to CHD in a context dependent manner while only a small number of variables were related to a specific outcome. We also observed that loss of complexity in the network was linked to a future CHD event. We propose that novel, non-linear, and integrative epidemiological approaches are required to combine all available information, in order to truly translate the new advances in medical sciences to gains in preventive measures and patients care.

## Introduction

In contrast to Mendelian disorders, complex diseases, such as Coronary Heart Disease (CHD), are the result of complex interactions between a great number of genetic polymorphisms, each with a small effect on risk, and a multitude of lifestyle and environmental factors and parameters [1]. Despite this, the methodology used to understand the genetic basis of the disease relies on the test of the association or linkage between a single nucleotide polymorphism (SNP) and an intermediate phenotype of CHD or the disease itself. The problem of multiple correlations between the risk factors is usually considered a hindrance and is not taken into account. Novel statistical models able to account for the patterns of interconnections between the different parameters affecting disease risk are required to better understand the mechanisms involved in the disease and identify targets for prevention and treatment. These kinds of complex models, able to consider all the correlations at the same time, are the basis of systems epidemiology.

Machine learning, the construction and study of algorithms able to use previous examples to provide accurate prediction in new data, has gained popularity in problems where the relationships between the variables of interest are very complex [2]. Artificial adaptive systems present an appealing method to address complexity [3,4]. Based on a set of simple rules, the system attempts to learn using some of the data, and apply its “knowledge” to the rest of the available information. Their main feature is the ability to modify their internal structure in response to the data presented [5]. Compared to the standard statistical methods used in epidemiology, these models are capable of analyzing all signals at the same time and to account for non-linear relationships between all the variables considered [6].

Here we present the application of an integrative mathematical approach, based on an artificial adaptive system, as a first step towards a systems epidemiology approach for CHD. Our main aims were to capture some of the complexity in the relationships between CHD associated phenotypes and genotypes, beyond the abilities of current reductionist approaches, and provide an illustration of the use of machine learning and graphical models for the understanding of complex diseases in a systems level.

## Methods

### Population sample and pre-processing

We used the Northwick Park Heart Study II, a prospective study of 3,012 middle aged men, and information recorded at baseline together, with the 35 genetic variables previously associated with one or more CHD related phenotypes in the same sample, reported elsewhere [7]. All SNPs were recoded as indicator variables. The continuous variables were arranged into tertiles, similar to the three genotypic classes used, and then also recoded as indicator variables. The analysis with CHD was restricted to participants with complete records for all the phenotypic variables, resulting in 102 cases and 150 randomly drawn controls to balance the case-control ratio and make the problem computationally efficient. Our final dataset included 10 circulating biomarkers associated with CHD, four variables for anthropometric measures also associated with the risk of disease and 37 genetic polymorphisms associated with the CHD intermediate phenotypes or the disease (Table 1).

**Table 1:** Names and abbreviations of phenotypes and polymorphisms included in the analysis.

Variable		Abr. used	Indicator variable selected		
			1	2	3
Smoking (yes/no)		smoking	yes		
Age		age		X	X
Body Mass Index		bmi	X	X	X
Systolic Blood pressure		sysbp		X	X
Diastolic Blood pressure		diabp	X	X	X
Triglycerides		tg	X		
Total Cholesterol		chol		X	X
Low Density Lipoprotein		ldl	X		X
High Density Lipoprotein		hdl	X	X	
Apolipoprotein B		apob		X	X
Apolipoprotein A		apoa	X	X	
Lipoprotein-associated phospholipase		lppla2			
C-reactive protein		crp		X	X
Factor VII coagulant activity		viic	X	X	
Fibrinogen		fib			
ALX homeobox 4 gene	rs729287	ALX4	X		
Angiopoietin-like 4 gene	E40K	ANGPTL4		X	
Apolipoprotein B gene	rs585967	APOB			
Apolipoprotein E gene		APOE	X	X	
Apolipoprotein-A5-A4-C3-A1 gene cluster	rs6589566	ApoA5-A4-C3-A1		X	
Arachidonate 5-lipoxygenase-activating protein gene	rs3885907	ALOX5AP	X		
Calpain 10 gene	rs4676411	CAPN10			
Cathepsin S gene	rs11576175	CTSS			X
Cholesteryl ester transfer protein gene	rs708272	CETP	X		
Coagulation factor VII gene	rs6046	F7	X		
Complement component 2 gene	rs7746553	C2			
Complement component 3 gene	rs344550	C3			
C-reactive protein gene	rs3093077	CRP	X	X	X
Cyclin-dependent kinase inhibitor 2A/B (Chr9p21)	rs10811661	CDKN		X	X
Exostosin 2 gene	rs3740878	EXT2	X		X
Fibrinogen alpha chain gene	rs4508864	FGA	X		X
Glucokinase (hexokinase 4) regulator gene	rs780094	GCKR	X		X
Glutathione S-transferase mu 3 gene	rs3814309	GSTM3	X	X	
Glutathione S-transferase mu 4 gene	rs1537236	GSTM4	X	X	
Hepatic lipase gene	rs1800588	LIPC	X	X	X
Insulin gene	rs689	INS			
Insulin-like growth factor 2 gene	1252T/C AluI	IGF2		X	X
Interleukin 1 receptor antagonist gene	rs397211	ILRN1	X	X	
Interleukin 18 receptor accessory protein gene	rs11465699	IL18RAP		X	
Interleukin 6 receptor gene	rs4075015	IL6R			
Lipoprotein lipase gene	rs301	LPL		X	X
Low density lipoprotein receptor gene	rs6511720	LDLR			
Low density lipoprotein receptor-related protein 5 gene	rs11602256	LRP5			X
Nitric oxide synthase 3 gene	rs3918232S3	NOS3	X		
Phospholipase A2, group VII gene	rs1051931	PLA2G7	X		X
Platelet/endothelial cell adhesion molecule gene	rs1131012	PECAM1	X	X	X
Proprotein convertase subtilisin/kexin type 9 gene	rs11591147	PCSK9		X	
Protein C receptor gene	rs867186	PROCR			X
Toll-like receptor 4 gene	rs11536857	TLR4	X		X
Transforming growth factor, beta 1 gene	rs4803455	TGFB1	X	X	
Uncoupling protein 2 gene	rs11602906	UCP2			
Uncoupling protein 3 gene	rs1685354	UCP3	X		X

The phenotypes were selected as established risk factors or markers of CHD and their associated polymorphisms. [7]. Only the single top SNP was included for each gene considered. Before analysis each SNP was recoded as three indicator variables. To maintain the three variables per genotype coding, the continuous phenotypes were transformed to tertiles (S1 Table). The full list of phenotypic tertiles and indicator variables used can be found in S2 Table. The three last columns show the generated indicator variables selected from the TWIST procedure as predictive of CHD. Out of the original 150 variables, 75 were retained. Continuous traits in tertiles and genotypes as three genotyping classes. In the abbreviations, index numbers after the gene names refer to common homozygote (1), heterozygote (2) and rare homozygote (3). The *APOE* gene polymorphism was coded so that 1 were the E2 carriers, 2 were those within the E3E3 category and 3 were the E4 carriers. Smoking is a dichotomous variables of yes or no smoking.

### Training with Input Selection and Testing (TWIST)

The TWIST protocol [8,9], a combination of artificial neural networks (ANNs) and a genetic algorithm for resampling and feature selection was used to select the smallest combination of indicator variables derived from optimizing the grouping of individuals compared to their observed CHD status in a testing set while making sure that the small number of observations does not affect the resampling process through an uncharacteristic subset selection. A number of learning methods, representing the main families of algorithms available, were applied on the training set in each successive iteration of the algorithm. The solutions better able to classify the individuals in future CHD cases were considered as fitter and they were overrepresented in the next generation (S1 Appendix).

### Graphical representation

Following variable selection, the indicator variables were arranged into an undirected tree graph, called the minimum spanning tree (MST) [8,10,11] using ANNs to optimize the links between the most similar indicator variables, and removing connections that produce loops in the graph (S2 and S3 Appendices). The Maximally Regular Graph (MRG) [12] including circular links between the indicator variables previously eliminated from the MST was also constructed. The complexity of the generated MRG was measured using an index of “hubness” named H, taking into account the presence of highly connected nodes [11], and a measure of topological entropy [13]. The Meta-MST, is a variant of the MST showing the most stable connections represented in 9 or more of the 10 MSTs constructed after randomly excluding 10% of the records. A more detailed description of the methods can be found in the appendix and supporting information.

## Results

### Variable Selection Using the TWIST Procedure

Table 1 shows all the variables considered during the variable selection procedure. The indicator variables used and their ranges are shown in S1 and S2 Tables. From the 150 indicator variables included in the analysis, 75 were retained as predictive of a CHD event (Table 1). The TWIST procedure used excluded a number of phenotypic categories, such as young age, no smoking (including both ex- and never-), the lowest tertiles of ApoB, total cholesterol, systolic blood pressure and CRP, and highest tertiles of ApoAI, factor VII complement, triglycerides and HDL. The intermediate tertiles of LDL and triglyceride levels were also not part of the selected indicator variables. All categories representing both Lp-PLA2 and fibrinogen proteins were removed during selection. None of the indicator variables for the *INS*, *IL6R*, *UCP2*, *C3*, *LDLR*, *CAPN10*, *APOB* and *APOC2* genes were selected. Other genotyping classes removed were the common homozygous categories for *IGF2*, associated with higher ApoAI, *CDKN2A* locus (Chromosome 9p21), associated with lower CHD risk, and *LPL*, associated with lower CHD risk traits, the rare homozygous categories for *PCSK9*, protective for CHD, the *GSTM4*, *ILRN1*, *TGFB1* and *GSTM3* genes, associated with higher CHD risk traits, and the heterozygous categories for *GCKR*, *EXT2*, the fibrinogen locus, *PLA2G7*, *UCP3*, and *TLR4*. Some loci were represented by a single genotypic class, the common genotype for *CETP*, *F7*, *ALX4*, and *ALOX5AP*, the heterozygote genotype for *IL18RAP*, and the *APOA5-A4-C3-A1* gene cluster, and the rare homozygote category for *PROCR*, *LRP5* and *CTSS*. The final list of factors retained can be seen in S3 Table while those removed are in S4 Table.

### The Auto Contractive Map

**Fig 1:**
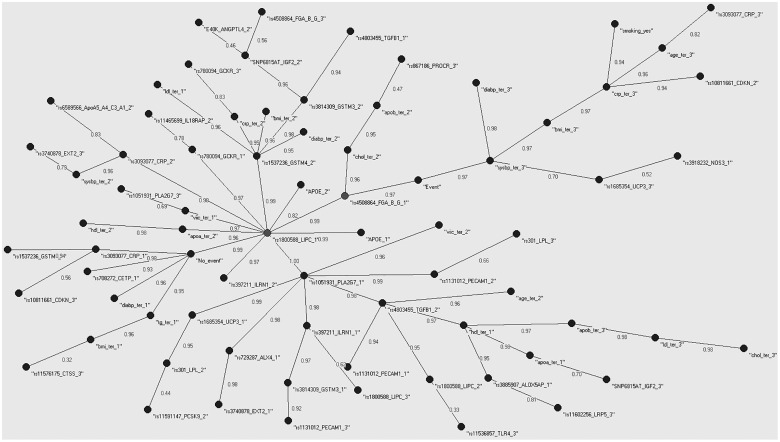
Minimum Spanning Tree (MST) for the TWIST selected variables. Minimum Spanning Tree (MST) for variables selected as informative for CHD by TWIST. Presence or absence of a CHD event during follow-up is included as two separate nodes in the tree. Only positive associations between the nodes, optimized for all other available connections, are represented in the graph. The numbers on the edges are a measure of similarity between the variables. Most risk factors considered are situated between the two nodes with paths able to reach either. A smaller number of parameters are characteristic for the Event or No_Event categories with their paths unable to reach one of the Event nodes without passing the other. Genotypes are coded as 1 for the common homozygotes, 2 for heterozygotes and 3 for the rate homozygotes. Phenotypes are in tertiles with 1 the tertile of lowest values.

**Fig 2:**
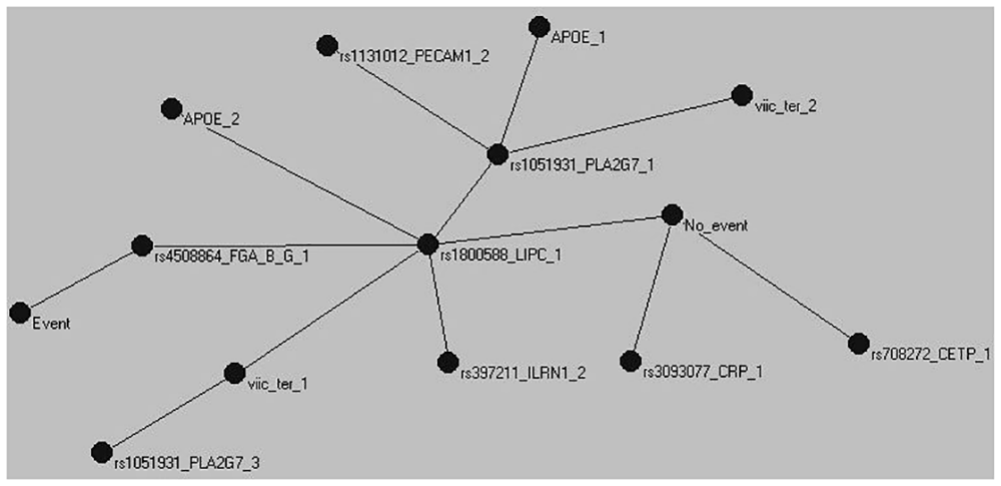
Meta-MST graph showing the connections represented in at least 9 of the 10 MSTs constructed after randomly excluding 10% of the records. In contrast to statistical testing, where small sample sizes lead to loss of statistical power for the identification of associations, the method used here is affected in terms of the stability of the proposed solution. Despite the smaller Meta-MST graph compared to the full MST, the main core of the graph with the two event nodes at opposite sides of the *LIPC* gene and fibrinogen locus nodes and most variables between them remains unchanged.

Following selection, we used the AutoCM to represent the main connections between the indicator variables. Fig 1 shows the MST for the TWIST selected indicator variables, CHD yes (“Event”) or no (“No Event”) and the structure of the connections between them. Both Event and No Event were nodes in the graph. The absence of an event was characterized by low diastolic blood pressure, triglycerides, and BMI, and a number of genetic polymorphisms, such as *CETP*, *CRP*, and *GSTM4* common homozygotes. The *CTSS* and *CDKN2A* loci were also found in the area but their correlation to the other nodes was weak. In contrast, presence of a CHD event within the follow up period was characterized by advanced age, smoking, high BMI, systolic and diastolic blood pressure, and CRP. Genetic polymorphisms present in this part of the tree included the rare homozygotes of *CRP*, and the heterozygotes of *CDKN2A*, while the *NOS3* and *UCP3* polymorphisms were weakly connected within this area of the network. Most of the indicator variables can be found somewhere between the Event and No_Event nodes, suggesting that these factors are not exclusive to *either* of the CHD categories. Two polymorphisms, one from the fibrinogen cluster and the other from the *LIPC* gene are situated on the path linking the two CHD states. The rs4508864 SNP of the fibrinogen beta chain gene is the center of the graph, the point with the minimum distance to all other nodes. The *LIPC* polymorphism on the other hand, is the central hub of the entire network with 11 branches radiating away from it. These highly connected nodes are important components of the model and can either coordinate, or be affected by, the processes around them, and organize the interactions between different parts of the system [14]. The Meta-MST supports the stability of the fibrinogen and *LIPC* genes as intermediates between event and no event and the importance of the *CRP* and *CETP* nodes to protection from CHD (Fig 2).

**Fig 3:**
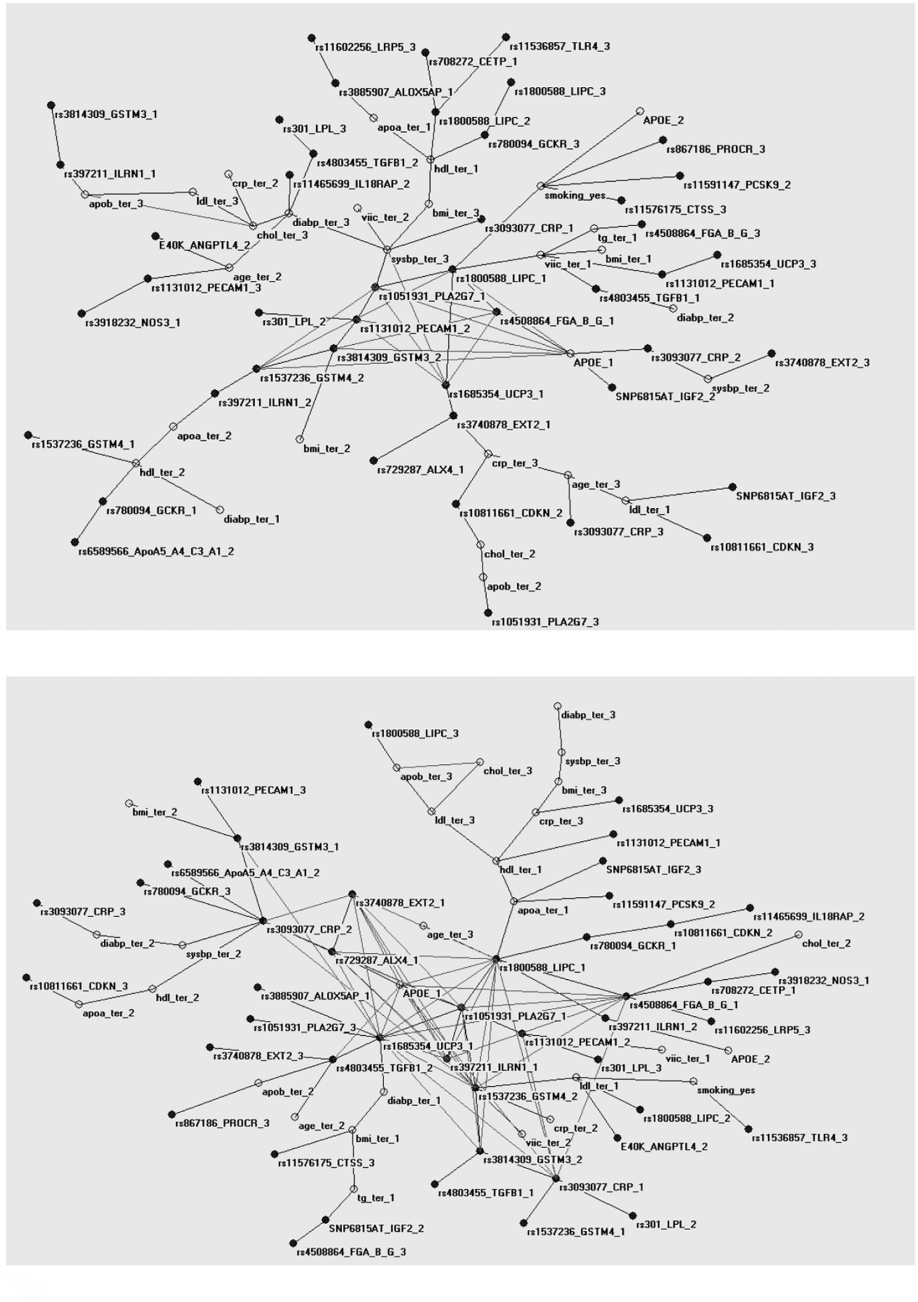
Maximally Regular Graph (MRG) for those that developed CHD and those that remained CHD free during follow-up. Separate Maximally Regular Graphs (MRG) for those that a) developed or b) remained free from CHD during the study 10 year follow-up. While the Minimum Spanning Tree (MST) represents the energy minimization state of all the correlations in the graph, mapping a single link between the variables and dropping all other links leading to cyclic structures. The MRG shows the maximum intrinsic complexity of the map by including the highest number of cyclic regular microstructures between the nodes. The graph for those that went on to have a CHD event has a smaller number of complex structures compared to the graph for those that remained healthy.

We constructed two further maps of the indicator variable set, for those that went on to develop CHD and those that did not (Fig 3). In this case we focused on the H index (H) and the topological entropy (E_G_) as measures of overall complexity of the network. For the part of the sample that went on to develop a CHD event (H = 0.373, E_G_ = 40.5), the MRG shows two distinct loops, one between the top tertiles of ApoB, total and LDL-cholesterol, and one between genotypes of the *PLA2G7*, *PECAM1*, *APOE*, *LIPC* and the fibrinogen cluster of genes. In comparison, the MRG of those free of CHD shows an increase of complexity as measured by the entropy but not the H index (H = 0.375, E_G_ = 42.6). The previously seen loops are still present, but now the connections between the SNPs are much more numerous than before. A number of additional polymorphisms in *CRP*, *GSTM3*, *GSMT4*, *GCKR*, *UCP3*, *ILRN1*, *ALX4*, and *EXT2* are now part of the central diamond-like structure of the MRG.

## Discussion

Using a number of CHD classical risk factors and genetic polymorphisms previously associated with them, we applied a feature selection procedure designed to account for non-linear associations between the variables. The selected indicator variables were then arranged in a map representing the connections between all the nodes at the minimum energy state of the network. The maximum complexity map was considered for cases and controls separately. We propose that modern machine learning approaches are able to provide information on the complex associations between risk factors, reveal the complexity in the system, and inform basic science on testable hypotheses.

### TWIST

Selection of the most informative variables from the original set was done using the TWIST system. All phenotypes were represented by at least a single category in the selected model except fibrinogen and Lp-PLA2. SNPs directly linked to plasma Fibrinogen (rs4508864) and Lp-PLA2 activity (rs1051931), were retained in the model. Both loci have been previously associated with a number of cardiovascular related phenotypes and diseases [15], suggesting that their correlation with CHD is likely due to pleiotropic effects through other, as yet unmapped, pathways. A number of parameters were also dropped from the final set of indicator variables, including no-smoking, young age, low ApoB, CRP and systolic blood pressure, and high ApoAI and HDL. Although, these indicator variables are statistically correlated with CHD, their removal from the final set and the inclusion of the opposite complementary feature was due to the redundancy of information between the two classes. In contrast, some of the variables considered maintain all indicator variables in the selected set, signifying the presence of interactions with other variables included. In non-linear systems opposite indicator variables can act differently according to the positive or negative sign of another indicator, explaining why some variables classes are not redundant as would be the norm in a linear setting.

For a number of genes, all three SNP genotypes were excluded from the model. Although some of these loci are well established risk factors for CHD, it is possible that the inclusion of phenotypes through which their effect is manifested leads to their removal. This is not unexpected since the genetic variants are more distant to the disease, compared to the intermediate traits they affect, and their CHD predictive ability can be fully accounted by them. For other SNPs, both homozygotes were removed from the model but not the heterozygotes. Over-dominance is possible for certain phenotypes, but it is usually considered as arising from chance when protein levels or action are considered, although marginal effects of interactions, or memberships of genes in pathways not included in the present analysis is possible.

Of course, a large number of feature selection methods have been proposed [16] making use of both theoretical and empirical criteria [17]. Here the TWIST system minimizes the classification rate within the confusion matrix, although other cost functions can be used, including the popular root mean square error. The key advantage of the method used is the resampling of the data in such a way that the assumption for the training and testing set are met, irrespective of the rather low number of individuals available.

### The Auto Contractive Map

The MST revealed that most of the indicator variables were between the two states of Event and No Event. Although these variables are correlated with subsequent presence or absence of an event, and this correlation can sometimes be high, the relationship is conditional on interactions or other complex processes operating in the organism [18]. The position of the nodes in the tree is based on the strength of their correlation to all other variables of the tree and how these arrange to produce single paths that link all the variables. Parameters situated between the two CHD nodes can reach either, with the weights along the path quantifying how favorable the path is. The parameters stemming away from the Event and No Event nodes have a direct path of association with future disease, sometimes through other related parameters, and this path can reach only one of the disease nodes without passing through the other.

Two nodes are directly on the path between the Event and No_Event categories, the fibrinogen beta chain gene and *LIPC*. Fibrinogen has been described as a possible factor linking vascular pathology with Alzheimer′s disease [19]. *LIPC* encodes hepatic triglyceride lipase and the variant typed is in the promoter of the gene and is believed functional by affecting transcription and thus gene expression [20]. Hepatic lipase is considered as a major factor in HDL metabolism and the remodeling of lipoproteins and their uptake by cell surface receptors [21]. The promoter variation included in the model has been suggested as both pro- and anti-atherogenic dependent on the underlying lipid profile [22] [23], which supports its position in the tree.

The common *CETP* homozygotes and the low diastolic blood pressure category, were directly associated with the No_Event node. The rare homozygotes of the *CTSS* gene are found in the same branch as the low level triglycerides and BMI categories. This gene, is coding for the cathepsin-S protein, implicated in vascular and metabolic complication of obesity [24], which our results suggest might also play a role in CHD protection.

Systolic blood pressure is central to the association of events with the other nodes, with branches linking it to diastolic blood pressure, BMI, and *UCP3*. The role of pulse pressure in CHD is well established [25]. The proximal node to *UCP3* is the *NOS3* gene, coding for the nitric oxide synthase-3 protein, which is believed to affect blood pressure through regulation of vascular tone [26]. Although no direct relationship between *UCP3* and *NOS3* has been observed, as proposed here, both are associated with sports performance [27,28] suggesting a common factor between the two and a link between potential athletic performance and health.

A branch stemming away from the high blood pressure and No_Event nodes contains a number of inflammation and stress response mechanisms. C-reactive protein (CRP) is an acute phase protein rising in response to inflammation or tissue damage. *GSTM4* codes for a Mu-Class glutathione S-transferase responsible for detoxification of electrophilic compounds [29], while the *CDKN2A* locus works as a cell growth regulator. Neither of the loci has been previously shown to function in association with *CRP*, although mRNA expression of inflammation-related genes in leukocytes might be able to upregulate *CDKN-1A*, at least in preeclampsia [30].

To summarize the MST findings, our results support the idea that low levels of TG, adiposity and diastolic blood pressure are associated with low risk, as are genes with a favorable response to stress. At the opposite end, high blood pressure, especially systolic blood pressure, and inflammation, or stress in more general terms, are closely related to a CHD event. Most lipids phenotypes, as well as a number of other factors, are situated somewhere between the two and their relationship with risk is conditional to other risk factors.

### The Maximally Regular Graph

Comparing the dynamics of those that went to develop heart disease to those that remained CHD free showed that the disease state was characterised by a previous loss of complexity as measured by the entropy of the graph. The H index though, was very similar between the two, suggesting that the difference of complexity seen was not due to changes of the graph hubs, but was based on a more general change in the distribution of connections. In addition to the measures used here a number of other methods to quantify structural complexity are available. These range from the most simple, such as counting the number of nodes and edges, to more complex measures, such as vertex degrees, shortest paths, or the more recent Eigenvalue-based measures [31–33]. Different measures of complexity do not always agree, even when they belong in the same general category [34], and the relationships between them are just starting to being fully explored [35]. Generally, there is no single best measure to describe complexity in a graph and the choice usually relies on the application.

In the MRG generated, those that went on to develop the disease show two simple loops, one between lipid phenotypes and one between genes involved in lipid metabolism and inflammation. Those that remained CHD free exhibit the same loops but much more complex, having multiple connections per indicator variable and an increased number of SNPs in the central complex structure. The results obtained suggest that we observe the loss of homeostasis. All living systems are balanced with interconnecting and frequently overlapping pathways. These networks carry the ability to respond to changes of state by attaining a new balance, or returning the system to its initial state through a series of complementary processes. Loss of this ability leaves the systems vulnerable to changes that are incompatible with normal function. The failure of the algorithm to find higher order structures within the data could indicate the break-down of the coordination of the system and the progressive vulnerability of the organism to disease.

### Limitations

A number of limitations are evident in our study. The biggest limitation was the low number of events considered. As the problem was approached through an optimization procedure, the small sample size does not affect our results in terms of statistical power but through the stability of the proposed solution. This is evident from the much smaller meta-MST compared to the original graph, showing only the portion of the MST that remained stable, in at least 9 of the 10 graphs we constructed by resampling. A larger, independent dataset will help with the stability of the inferred networks and selection, removing some of the noise and any chance links that might have been included in the graph.

### Conclusions

The shift of focus from isolating single causes of disease to the use of complex dynamic models able to account for the disease at multiple levels of organisation is challenging the current paradigm of epidemiology, and points towards more integrative methods of a systems epidemiology approach [36]. Although we focus on CHD, other cardiometabolic diseases, such as type 2 diabetes and stroke, could be potentially included in such models [37] in order to study the interactions between them and their common pathways. The use of complex systems models in epidemiology has been used successfully in infectious diseases source identification [38], susceptibility, and transmission [39,40], but still has limited application in non-communicable age related complex diseases. Recent advances on the organisation of complex networks will significantly help our understanding and interpretation of these models [41], and more importantly, how changes in a specific biological processes cascade to a disease phenotype and ultimately a CHD event [42]. Our results suggest that this kind of approach can provide novel insights into the problem of non-communicable age related complex diseases such as 1) the ability to concisely describe the associations of all risk factors between them and with the disease, 2) the context-dependent link between most risk factors with the disease state and 3) the loss of network complexity prior to a CHD event. We believe that this approach will contribute to a better understanding of CHD and provide new testable hypothesis.

## Additional Methods

### Study Design and Phenotypic Measures

The Northwick Park Heart Study II is a prospective study of 3 012 middle aged men, sampled from nine UK general practices between 1989 and 1994. All men were free from disease at the time of recruitment and aged between 50–64 years[43]. Information on lifestyle habits, BMI, blood pressure, and more than 15 circulating blood factors associated with CHD risk were recorded at baseline, and for some, on subsequent prospective follow-ups. DNA was obtained at the time of recruitment from 2 775 men. In the first 10 years of follow-up, 296 definite fatal or non-fatal CHD events had occurred. Details of recruitment, measurements, follow-up and definitions of incident disease have been reported elsewhere [43]. The study was approved by a UCL review committee and the subjects gave informed consent.

### Genotyping

A customized Illumina 768 SNP genotyping array was assembled to comprehensively capture common genetic variation in more than 76 genes involved in pathways linked to CHD risk as described previously [7]. These were supplemented with information on 173 SNPs in a further 82 genes previously typed in this data set. Missing genotypes were imputed using PHASE with the most likely genotype considered as the true genotype for the individual [44].

### Associations and Data Recoding

After filtering for deviation from Hardy Weinberg equilibrium (p<5x10^-5^) and minor allele frequency 0.1%, 614 SNPs were tested for associations with 12 blood biomarkers of which six were protein phenotypes (CRP, fibrinogen, apoAI, apoB, Lp-PLA2 and factor VII) and six were non-protein metabolic phenotypes (total-cholesterol, HDL- and calculated LDL-cholesterol, triglycerides, homocysteine and folate), identifying 140 SNP-phenotype associations in 37 different genetic loci [7]. Testing for independence between the signals, we found that the number of independent signals was very close to the number of genes in which the signals were located [7]. For simplicity, and to avoid the fragmentation of information per gene locus, we kept a single SNP per locus even if multiple SNPs showed evidence for independent associations. Therefore, the number of signals was reduced by selecting only the SNP with the smallest p-value of each locus, giving us a total of 37 genotypic variables. Full description of the association between all the available polymorphisms and the phenotypes considered can be found in Drenos et al 2009 [7].

All SNPs were subsequently recoded as indicator variables, thus generating three new variables for each SNP. Similarly, the continuous phenotypic variables were first arranged into tertiles, to correspond to the three genotypic classes used, and then also recoded as indicator variables of low intermediate and high levels of the trait with 1 denoting membership of the individual in this category and 0 otherwise. The choice of coding was based on the properties of the algorithms used and the specific features of the graph that we wanted to emphasize. The analysis with CHD was restricted to those participants with complete records for all the phenotypic variables considered, thus giving us a set of 102 cases (mean age = 56.96 25–75% = 54–60 years of age) and a randomly drawn subset of 150 controls (mean age = 55.8 25–75% = 53–59 years of age) for a more balanced dataset.

## Analysis

### TWIST

All analysis was completed using algorithms and applications developed by Semeion Research Centre of Sciences of Communication in Rome. To include only the most informative of the available variables we used a genetic algorithm, called the Genetic Doping Algorithm [45], which uses the principles of evolution to optimize the training and testing sets and to select the minimum number of variables capturing the maximum amount of available information in the data. Contrary to statistical linear models using indicator variables, TWIST does not require the omission of a reference category. This is due to the focus of the artificial neural network on prediction rather than estimation. If some of the indicator variables can completely account for the predictive ability of the others, those will be excluded by the algorithm during the selection process. The method is called the TWIST protocol and has been previously applied successfully in similar problems [8,9]. The advantages of the approach are the sub-setting of the data in two representative sets for training and testing, which is problematic in small datasets, and the use of a combination of criteria to determine the fit of the model. TWIST is comprised of two systems, the T&T for resampling of the data and the IS for feature selection, both using artificial neural networks (ANNs). The T&T system splits the data into training and testing sets in such a way that each subset is statistically representative of the full sample. This non-random selection of subsets is crucial when small samples are considered and the selection of non-characteristic and extreme subsets is likely. The training phase is making use of a combination of 13 learning machines representing the main methodologies in the field including logistic regression, random forests, support vector machines and naïve Bayes (a full list can be found in S1 Appendix). The IS system uses the training and testing subsets produced to identify a vector of 0s and 1s, describing the absence or presence of an indicator variable, that is able to optimize the categorization of the individuals in cases and controls compared to their observed CHD status. For this, a population of vectors, with each vector a combination of the indicator variables, is allowed to “evolve” through a number of generations in order optimize the prediction of CHD, as a natural population evolves to optimize fitness under a specific set of environmental conditions. The vectors with the best predictive ability are overrepresented in the next generation while a smaller number of sub-optimal vectors are maintained to give rise to the following generation. Some instability, in the form of low predictive ability vectors, is introduced in the process to avoid the problem of finding a solution which is optimal under a narrow set of conditions, also known as a local optimum. This step ensures that the attributes do not include redundant information or noise variables that will decrease the accuracy of the map and increase both the computing time and the amount of examples necessary during learning. In addition, feature selection permits the easier interpretation of the graph of relationships between the variables in respect to their arrangement compared to the CHD nodes. The TWIST approach is described in detail in S1 Appendix

### Auto-CM

Following feature selection, the indicator variables considered as informative were arranged into an undirected map of connections using an adaptive model, the Auto Contractive Map (AutoCM) [8,10,11]. The AutoCM produces a matrix of the correlations between the variables in the dataset, including information on the complex dynamics of adaptive interactions in the form of weights (w) between the variables [10]. In this case correlation does not refer to a linear relationship between the variables, as in the Pearson product-moment correlation, but to the similarity between the binary vectors of the indicator variables described as the degree of membership of the two nodes in the same fuzzy set. The relationship between the two variables can be said to be null or very low when w ≤.33, quite low when 0.33 < w ≤ 0.66, quite high when 0.66 < w ≤ 0.84, and very high when w > 0.84. Based on this definition of correlation, only positive associations between the nodes are presented in the graphs. Starting from a connected, weighted graph, linking all variables to all others, the algorithm constructs a tree with variables as nodes and their distances as edges, called the minimum spanning tree (MST) [46]. This includes all the variables considered, linked in a way that the energy structure of the system is minimized, or if weights are correlation measures, in a way that these are maximized for all the connections in the graph [47,48]. The MST algorithm does not map all the correlations present in the data but only the simplest view of the strongest correlations present between the nodes in the system in a way that all the nodes are connected to another node and only a single path is available to travel between two nodes (no loops). Also, each connection between nodes is adjusted for all the other connections between all the nodes included in the graph through an optimization of the square matrix containing all correlations between the indicator variables. In the MST, every link able to generate a cycle into the graph is eliminated, irrespective of its strength of association, resulting in a simplified graph [11]. All three algorithms for graphs construction are based on the architecture of ANNs with a number of unique features. A detailed description of the algorithm and its comparison with other methods can be found in S2 and S3 Appendices.

### MRG

The Maximally Regular Graph (MRG) [12] is the graph of highest complexity among all the graphs generated during calculation of the MST. With the MRG, links, previously removed from the MST, are now re-introduced in the graph. The number of these new links is proportional to the overall number of associations between the variables and hence an indicator of its overall complexity. In this case the complexity is measured though the H function reflecting the “hubness” of the graph (S4 Appendix) and a topological entropy taking into account the mean topological complexity, as the ratio of the number of the arcs to the number of the pruning cycles necessary to delete the graph, and the information contribution of each node to the global information of the graph [11]. The MRG is built in a process that maximizes the number of added connections while maintaining the structure of the original tree and produces structures where each node has the same number of other connected nodes (details in S4 Appendix).

### Meta-MST

The Meta-MST, is a variant of the MST showing the most stable connections represented in at least 9 out of 10 times the MST was reconstructed after randomly excluding 10% of the records.

## Supporting Information

S1 Appendix Training with Input Selection and Testing (TWIST). (DOCX)

S2 Appendix Auto Contractive Map (Auto-CM). (DOCX)

S3 Appendix Auto-Contractive Maps and MST: an application to the toy Database. (DOCX)

S4 Appendix The Maximally Regular Graph (MRG). (DOCX)

S1 Table Range of tertiles for the continuous phenotypes used. (XLSX)

S2 Table Full list of indicator variables used in the TWIST feature selection procedure. Genotypes as indicator variables of the three genotypes. Phenotypes as tertiles of levels when not categorical.
(XLSX)

S3 Table List of indicator variables selected in the best subset of variables by the TWIST procedure. The evolutionary algorithm of TWIST was run for 500 generations.
(XLSX)

S4 Table List of indicator variables removed from best fitted model after selection by the TWIST procedure. The evolutionary algorithm of TWIST was run for 500 generations.
(XLSX)
