# Supplementary material for: Networks in Coronary Heart Disease Genetics As a Step towards Systems Epidemiology
Source: PLoS One. 2015 May 7;10(5):e0125876. doi: 10.1371/journal.pone.0125876 (PMC4423836; doi:10.1371/journal.pone.0125876)
Supplement: S1 Appendix — (DOCX) [file pone.0125876.s001.docx]

Appendix 1

**Training with Input Selection and Testing (TWIST)**

**Massimo Buscema**

Validation protocol and Input selection are some of the most crucial problems in Pattern Recognition for machine learning. The two problems can be defined as:

1. How to generate an optimal pair of training and testing sets statistically representative of the assigned problem;
2. How to select the minimum number of input features able to maximize the accuracy of the dependent variables (target), in a blind test.

We propose the TWIST algorithm (Training With Input Selection and Testing) as a possible method to answer these two questions.

Training & Testing Optimization (T&T)

We can distinguish different types of cross-validation procedures: k fold cross-validation, leave one out, boosting, 5x2 cross-validation, training and testing set splitting, and others (DIETTERICH 1998). All these procedures represent different statistical strategies to generate tasks for machine learning training and testing. Any single distribution of the source dataset in a training set and in a testing and/or a validation set is always executed with a random splitting of any single record (observation) of the source dataset in order to generate two subsets of data more or less equivalent to each other, from a statistical point of view. In fact, the random criterion aims to optimize the following function:

1. *f_1_*($d_{\Gamma}^{[tr]}$)*f_2_* ($d_{\Gamma}^{[ts]}$)*f_0_* ($d_{\Gamma}^{[global]}$)

where *f_1_*($d_{\Gamma}^{[tr]}$) and *f_2_* ($d_{\Gamma}^{[ts]}$) = probability density function of testing and training subset, respectively; *f_0_* ($d_{\Gamma}^{[global]}$) = probability density function of the global dataset. This means that the random criterion aims to generate two subsets with, more or less, the same probability density function, with each one of these subsets statistically equivalent to the global dataset.

The random criterion tries to approximate the cost function defined in the equation (1). But to optimize this cost function we should consider every possible combination of each record into the two subsets and then for any combination to measure and to compare the probability density function of each subset. There is no evidence that the random criterion can optimize this cost function.

Now, given a dataset *D* _Γ_ of N records, the number of samples *d_Γ_* which are comprised of K possible records is given by:

$$\left(\begin{matrix} N \\ K \end{matrix}\right)$$

Varying K, you have:

(2) $\sum_{K=0}^{N} \left(\begin{matrix} N \\ K \end{matrix}\right)=2^{N}$ (*)

(*) the search space is 2^N^, but the acceptable space is $\frac{2^{N}-2}{2}$.

Therefore, a pair of training and testing sets represents, on the solutions space, a possible solution$x=\left(D_{\Gamma}^{[tr]},D_{\Gamma}^{[ts]}\right)$, given by the vector:


The problem described in equations 1-3 is a problem of operation research. To optimize the function presented in equation 1, we propose an evolutionary algorithm whose population expresses, after each generation, a different hypothesis about the splitting of the global dataset into two subsets. In each generation, each individual of the genetic population proposes which records of the global dataset have to be clustered into the subset A and which one into the subset B. Each individual of the genetic population is a vector of N Boolean values (1 or 0), where N is the number of the records of the global dataset. For this we used two independent Supervised Neural Networks (SNNs), typically back propagation based multilayer perceptron (MLP) models. The fitness for each individual of the genetic population is evaluated using the following five steps:

1. The first SNN (SNN_A) is initialized and trained using the subset A, and it is stopped when the training error (that is, for example, the RMSE) is minimized;
2. The SNN_A, with fixed trained weights, is applied in a blind way on the subset B, and its accuracy is saved;
3. The SNN_B (completely independent from the SNN_A) is initialized and trained using the subset B, and it is stopped when the training error (that is, for example, the RMSE) is minimized;
4. The SNN_B, with the trained weights fixed, is applied in a blind way on the subset A, and its accuracy is saved;
5. The minimum value of the two accuracies is assigned as fitness of the hypothesis of splitting, generated by any individual of the genetic population.

The steps from 1 to 5, named “Fitness Evaluation”, are executed for each individual of the genetic population, at any generation of the evolutionary algorithm.

The flow chart of the whole algorithm is as follows:

1. Genetic population initialization
2. Evolutionary loop
   1. Fitness Evaluation of the hypothesis of splitting of each individual of the genetic population at the generation (n) (From step 1 to step 5);
   2. Crossover and offspring generation;
   3. Random mutation is applied;
   4. Setup of the new population;
   5. If the average fitness increases the process starts from the beginning; otherwise is terminated;
3. Save the subset A and the subset B with the best fitness.

The advantages of T&T algorithm are:

1. The evolutionary algorithm used is a special enhancement of the classic genetic algorithm. Its name is Genetic Doping Algorithm (GenD). GenD has shown to be more effective than the classic genetic algorithm in many optimization problems (BUSCEMA 2004);
2. The multilayer back propagation, using the SoftMax algorithm (BRIDLE 1989) for classification tasks, is a very robust and fast artificial neural network (ANN). The back propagation SNN is also able, with a suitable number of hidden units, to compute any continuous function (RUMELHART 1986, CHAUVIN 1995);
3. The T&T algorithm uses all the information present in the global dataset. The optimized Subset A and Subset B can be used both for Training and Testing (learning from subset A and evaluate using subset B, and vice versa), with different learning machines (Neural Networks, Decision Trees, Bayesian Networks, etc.).

Comparison of the random criterion to setup the training and the testing sets has shown that the T&T algorithm is out performing the classic method in real medical applications (BUSCEMA 2005, PENCO 2005, GROSSI 2007a, GROSSI 2007b, LAHNER 2008, BUSCEMA 2010, ROTONDANO 2011).

Input Selection (I.S.)

Another significant methodological problem related to the application of machine learning to real databases of a large number of variables is which small set of variables provides the largest possible amount of information. In these conditions the *input space*, determined by all the possible combinations of the values of the observed variables, becomes so large that any research strategy to find the best solutions becomes very cumbersome when specific problems are tackled, e.g. classification tasks. It is important to carry out a preliminary analysis of the variables of the dataset since these can have different relevance in regards to the data mining that one intends to carry out. Some of the attributes may contain redundant information which is included in other variables, or confused information (noise), or may not even contain any significant information at all and be completely irrelevant. Therefore a procedure that will identify and select, from the global set, a subset consisting of those variables that are most informative towards the representation of the input patterns is necessary when dealing with classification problems solved with induction algorithms. Moreover, the accuracy of the procedure, learning time and the amount of examples necessary depend upon the choice of variables.

Feature selection techniques (also known as Subset Attribute Selection or Input Selection) were developed to determine which variables (or attributes) are most relevant in the representation of an input pattern, starting from a large data set. Feature selection extracts from a given dataset $D_{\Gamma}$ of M characteristics the best subset consisting of K characteristics. The number of possible subsets of K characteristics is given by$\sum_{K=1}^{M} \left(\begin{matrix} M \\ K \end{matrix}\right)=2^{M}-1$, and the best subset is the one that maximizes the generalization of the machine learning in the test phase. When the dataset has a high number of variables, an exhaustive search on the global set of characteristics is not possible. In this cases the techniques that can be used are based on a blind (Depth First) or heuristic search (Hill Climbing, Best First), although evolutionary search techniques have also been proposed (KUDO 2000; SIEDLECKI 1989).

Machine learning techniques can be developed using two different general approaches based on whether the selection of the variables is carried out dependently or independently of the learning algorithm used to build the inductor. The filter approach attempts to select the best attribute subset by evaluating its relevance based on the data. The “wrapper” approach, instead, requires that the selection of the best attribute subset takes place considering as relevant those attributes that allow the induction algorithm to generate a more accurate performance (JOHN 1994). The Input Selection algorithm (I.S.) operates as a specific evolutionary wrapper system that responds to the need to reduce the dimensionality of the data by extracting the minimum number of variables necessary to control the “peaking” phenomenon and, at the same time, conserving the most available information.

The implementation of the I.S. algorithm is very similar to what was described previously for T&T. In this case, each individual of the genetic population is a vector of M components of Boolean values, where M is the number of all the input variables of the global dataset. When the value of a component of the vector is 1 the corresponding input feature is saved, while if the value is 0, then the corresponding input feature is removed.

Training With Input Selection and Testing (TWIST)

To integrate our IS algorithm with the T&T algorithm in only one procedure we have to modify the structure of each individual of the genetic population, that we have already described. We have named this new algorithm TWIST (Training With Input Selection and Testing, see Buscema 2013 for updated details). In the TWIST algorithm every individual of the genetic population will be defined by two vectors of different length:

1. The first one, testing which records (*N*) has to be stored into the subset A and which ones have to be stored into the subset B;
2. The second one, testing which inputs (*M*) have to be used into the two subsets and which one have to be deleted.

After this modification the TWIST algorithm works as well as the T&T algorithm, already described. At the end of its evolution TWIST will generate two similar subsets of data with the minimal number of effective variables for pattern recognition. Figure 1 is a synthetic representation of the dynamics of the TWIST algorithm.

To test if the algorithm is overfitting the data to produce better estimates we generated a random dataset with the dependent variable randomly distributed. There are two possible outcomes of this test:

1. TWIST shows **better** results than using sub samples generated by means of a random splitting and K-Fold cross validation;
2. TWIST shows **the same** results as using sub samples generated by means of a random splitting and K-Fold cross validation;

TWIST can be considered as an appropriate method only when 2 is true and the algorithm behaves as a random strategy when there is no information available in the dataset. Consequently, any increase in the ability of the algorithm to extract useful information in datasets, when this information is really present, cannot be attributed to overfitting.

To test this hypothesis we have generated a dataset of 1000 records, each one composed of 20 random input variables and one random label as dependent variable. Then we processed the dataset using 4 different strategies:

1. A K-Fold cross validation (K=10);
2. A random splitting of the dataset into two subsets, to be used both as training set and testing set;
3. The T&T strategy to generate two subsets of 500 individuals to be used both as training set and testing set. Each back propagation was trained for 200 epochs. T&T run for 1000 generations
4. The TWIST strategy to generate two new subsets of 500 individuals with a minimal number of variables to be used both as training and testing sets. Again the back propagation was trained for 200 epochs. TWIST run for 1000 generations.

We used 13 different learning machines, representing the different main “families” of algorithms in the literature.. The list of the machine learning algorithms used is (WITTEN 2005, DUDA 2001, KUNCEVA 2004, ROKACH 2009):

1. Bayes Net;
2. Naïve Bayes;
3. Support Vector Machine (SVM);
4. Logistic Regression;
5. Multilayer Perceptron;
6. Sequential Minimal Organization (SMO);
7. IBk;
8. K Star;
9. Ada-Boost M1;
10. Logit Boost;
11. Rotation Forest;
12. J48;
13. Random Forest.

The algorithms were implemented using the WEKA software package (HALL 2009)

| **Accuracy in blind testing** | | | | |
| --- | --- | --- | --- | --- |
| **Weighted Mean** | **K-Fold (K=10)** | **Random Splitting** | **T&T** | **TWIST** |
| AdaBoostM1 | 48.70% | 48.10% | 51.76% | 52.22% |
| BayesNet | 51.90% | 51.90% | 47.61% | 51.92% |
| IBk | 49.60% | 50.40% | 50.00% | 50.77% |
| J48 | 51.90% | 51.90% | 47.61% | 51.92% |
| KStar | 51.20% | 50.70% | 49.79% | 53.50% |
| Logistic | 49.20% | 51.30% | 51.86% | 52.50% |
| LogitBoost | 49.50% | 47.90% | 51.72% | 51.80% |
| Multilayer Perceptron | 50.90% | 52.41% | 51.59% | 50.67% |
| NaiveBayes | 48.90% | 49.90% | 51.72% | 51.79% |
| RandomForest | 50.50% | 51.40% | 49.52% | 50.73% |
| RotationForest | 51.60% | 52.31% | 50.18% | 52.53% |
| SMO | 50.80% | 51.50% | 51.04% | 53.10% |
| SVM | 51.80% | 51.20% | 50.33% | 52.02% |
| **Average** | **50.50%** | **50.84%** | **50.36%** | **51.96%** |
| **Standard Deviation** | **0.0119** | **0.0145** | **0.0148** | **0.0087** |

Table 1: the results of the comparison.

The comparison suggests that T&T and TWIST are no different from random splitting and k-fold validation when no information is present in the data. We can conclude that in classification problems T&T and TWIST:

1. Do not code noise to reach optimistic results;
2. Are suitable algorithms to generate couples of subsets with similar probability density function;
3. Are valid strategies to allow machine learning to extract the most useful information form a dataset.

In the current application of the algorithm we used back propagation with self-momentum and a learning rate=0.1. The SoftMax algorithm was used for the output layer and the neural network included a layer of 12 hidden units.

Figure 1: Flow chart of TWIST algorithm

**References**:

DIETTERICH, 1998:

T G Dietterich, 1998, *Approximate statistical tests for comparing supervised classification learning algorithms*, Neural Computation, 10, (7), 1885-1924.

BUSCEMA, 2004:

M Buscema, *Genetic doping algorithm (GenD): theory and application*, Expert Systems, May 2004, Vol. 21, No 2, pp 63-79, Blackwell Publishing.

BRIDLE, 1989:

J. S. Bridle, *Probabilistic Interpretation of Feedforward Classification Network Outputs, with Relationships to Statistical Pattern Recognition*, in F. Fogelman-Soulié, and J. Hérault (eds.), *Neuro-computing: Algorithms, Architectures*, Springer-Verlag, New York.

CHAUVIN, 1995:

Y. Chauvin, and D. E. Rumelhart, (Eds.) *Backpropagation: Theory, Architectures, and Applications*, Lawrence Erlbaum Associates, Inc. Publishers 365 Brodway- Hillsdale, New Jersey, 1995.

RUMELHART, 1986:

D. E. Rumelhart, G. E. Hinton, and R. J. Williams, *Learning Internal Representations by Error Propagation*, in D. E. Rumelhart, and J. L. McClelland, (eds.) *Parallel Distributed Processing*, Vol.1 *Foundations, Explorations in the Microstructure of Cognition*, The MIT Press, Cambridge, MA, London, England 1986.

BUSCEMA, 2005:

Buscema M., Grossi E., Intraligi M., Garbagna N, Andriulli A. And Breda M., 2005, *An optimized experimental protocol based on neuro-evolutionary algorithms. Application to the classification of dyspeptic patients and to the prediction of the effectiveness of their treatment.* Artificial Intelligence in Medicine, 34, 279-305.

PENCO, 2005:

S Penco, E Grossi et al., 2005, *Assessment of the Role of Genetic Polymorphism in Venous Thrombosis Through Artificial Neural Networks* , in Annals of Human Genetics (2005) 69,693–706, University College London.

GROSSI 2007a:

E Grossi, A Mancini, M Buscema, 2007, *International experience on the use of artificial neural networks in gastroenterology*, in Digestive and Liver Disease 39 (2007) 278–285.

GROSSI 2007b:

E Grossi, M Buscema, 2007, *Introduction to artificial neural networks*, European Journal of Gastroenterology & Hepatology 2007, 19:1046–1054.

LAHNER 2008:

E Lahner, M Intraligi, M Buscema, et al., 2008, *Artificial neural networks in the recognition of the presence of thyroid disease in patients with atrophic body gastritis*, World J Gastroenterol 2008 January 28; 14(4): 563-568.

BUSCEMA 2010:

M Buscema, E Grossi, M Capriotti, C Babiloni, PM Rossini, 2010, *The I.F.A.S.T. Model Allows the Prediction of Conversion to Alzheimer Disease in Patients with Mild Cognitive Impairment with High Degree of Accuracy*, Current Alzheimer Research, 2010, 7, 173-187.

ROTONDANO 2011:

G Rotondano, L Cipolletta, E Grossi, 2011, *Artificial neural networks accurately predict mortality in patients with nonvariceal upper GI bleeding*, GASTROINTESTINAL ENDOSCOPY Volume 73, No. 2 : 2011.

KUDO 2000:

M Kudo, J Sklansky ,2000, *Comparison of algorithms that select features for pattern classifiers,* Pattern Recognition, 33 (1), 25-41.

SIEDLECKI 1989:

W Siedlecki, J Slansky, 1989, *A note on genetic algorithms for large scale on feature selection*, Pattern Recognition Letters, 10, 335-347.

JOHN 1994:

G John, R Kohavi, K Pfleger, 1994, *Irrelevant features and the subset selection problems*, In 11^th^ International Conference on Machine Learning, 121-129.

HALL 2009:

Hall, M, Frank, E, Holmes, G, Pfahringer, B, Reutemann, P, Witten, I H, 2009, *The WEKA Data Mining Software: An Update*, SIGKDD Explorations, Volume 11, Issue 1.

WITTEN 2005:

Witten, I H and E. Frank, E, 2005*, Data Mining*, Morgan Kaufmann.

DUDA 2001:

Duda, R O, Hart, P E, and Stork, D G, 2001, *Pattern Classification*, Wiley & Sons.

KUNCHEVA 2004:

Kuncheva, L I, 2004, *Combining Pattern Classifiers: Methods and Algorithms*, John Wiley and Sons, Inc.

ROKACH 2009:

Rokach, L, 2009, *Taxonomy for characterizing ensemble methods in classification tasks: A review and annotated bibliography*, Computational Statistics and Data Analysis 53, pp 4046-4072.

BUSCEMA 2013:

M Buscema, M Breda, W Lodwick, *Training With Input Selection and Testing (TWIST) algorithm: a significant advance in pattern recognition performance of machine learning,* in Journal of Intelligent Learning Systems and Applications, 2013, 5, 29-38.
