# Supplementary material for: Networks in Coronary Heart Disease Genetics As a Step towards Systems Epidemiology
Source: PLoS One. 2015 May 7;10(5):e0125876. doi: 10.1371/journal.pone.0125876 (PMC4423836; doi:10.1371/journal.pone.0125876)
Supplement: S3 Appendix — (DOCX) [file pone.0125876.s003.docx]

**Appendix 3**

**Auto-Contractive Maps and MST: an application to the toy Database**

**By Massimo Buscema**

In this section we will describe the application of the MST to the GANG database in order to compare the matrices of the distances generated by Auto-CM with those from other algorithms*.*

The Gang database is made up of 27 records, each identified with a name, and 5 variables corresponding to each subject’s characteristics. The Gang database appears as follows:


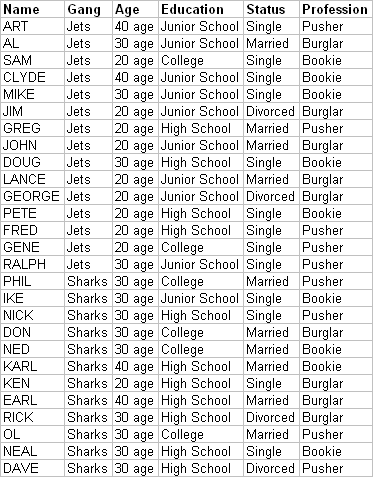


Table 1. Gang database.

To be able to process the database, that is, to transform it into a matrix of values, some pre-processing operations are needed.

The database presents 5 variables, each formed by different options:

- Gang = {Jets, Sharks};

- Age = {20’s, 30’s, 40’s };

- Education = {Junior School, High School, College};

- Status = {Married, Single, Divorced};

- Profession = {Pusher, Bookie, Burglar}.

We will transform all the options of each variable into new variables of a binary type (value 1 when the characteristic is present, 0 when that characteristic is absent) and each of them will be independent from all the others.

After this transformation we obtain a new database formed by 14 variables (Gang (2) + Age (3) + Education (3) + Status(3) + Profession (3)) and 27 records. In table 2 is the database after the transformation:

Table 2. Gang database after the transformation in 14 variables

At this point we have transposed the database and obtained a new database where records become variables and vice-versa, formed by 27 variables and 14 records:

Table 3. Gang database after the transposition

Both CM and a number of other algorithms have been applied to this new database in order to generate matrices of distances comparable with each other, in particular:

- Contractive Map (CM)
- Back Propagation (BP)
- Prior Probability (PP)
- Linear Correlation (LC)
- Euclidean Distance (ED)

Once the matrices of distances have been generated, the matrix of the adjacent boroughs from which to generate the MST graph was calculated. In this way, we obtained an MST graph for each algorithm.

**Results**

**Euclidean Distance**

By observing the MST graph in figure 1 the following considerations can be drawn:

- clustering is 91.7% correct (2 errors); in fact, KEN and IKE, both Sharks, in the graph are far from the other members of the same gang and linked to the two Jets who most match them; in particular, KEN is the only 20’ amongs the Sharks and is linked to PETE with whom he shares three characteristics (20’, High School and Single); IKE is the only one with Junior School among the Sharks and is linked to MIKE, with whom he shares the same characteristics, excluding the gang;
- the boundary line of the gangs is between DOUG and NEAL; in fact, except the gang, they have the same characteristics;
- a number of Jets who have the Burglar and Junior School characteristics in common (GEORGE, JIM, JOHN, AL and LANCE) are very close and linked to each other;
- all the couples with identical characteristics (PHIL-OL for the Sharks; LANCE-JOHN and GEORGE-JIM for the Jets) are directly linked to each other;
- PHIL and MIKE have a greater power of clustering;
- PHIL forms a hub with DON, OL and NED, who are the Sharks with the COL characteristic;
- MIKE forms a hub with IKE, RALPH and CLYDE, who are the Jets with the Junior School and Single characteristics;
- PHIL and MIKE have a greater power of clustering and, together with PETE, the greatest number of links (4);
- whereas DOUG and PETE have the highest protection grade and are at the centre of two ramifications.

Figure 1. MST graph resulting from the Euclidean distances matrix

**Linear Correlation**

The graph in figure 2 is very similar to the Euclidean Distance graph, with some minor differences:

- in this graph clustering is 95.8% correct; KEN is still far from the Sharks, but in this case he is linked to FRED, with whom he shares all three characteristics in common, as he does with PETE (20’, High School and Single);
- also in this case DOUG and NEAL are at the boundary line;
- this time IKE is linked to NEAL, with whom he shares two characteristics in common (Single and Bookie);
- in this graph the Jets sharing the Burglar and Junior School characteristics (GEORGE, JIM, JOHN, AL and LANCE) are also very close to each other and linked;
- all the couples with identical characteristics are directly linked (PHIL-OL for the Sharks; LANCE-JOHN and GEORGE-JIM for the Jets);
- in this case PHIL also has the greatest power of clustering and forms a hub with DON, OL and NED, who are the Sharks with the COL characteristic;
- MIKE forms a hub with IKE, RALPH and CLYDE, who are the Jets with Junior School and Single characteristics;
- the greatest protection grade belongs to DOUG and NEAL;
- the maximum number of links is 3.

Figure 2. MST graph resulting from the Linear Correlation distances matrix.

**Prior Probability**

This graph in figure 3 has characteristics similar to the preceding two, with some differences:

- in this graph clustering is 79.2% correct, this is noticeable of the break-up of the Jets’ cluster into three ramifications: two depend on DOUG and one on DON;
- IKE is far from his gang and is linked to MIKE (as per Euclidean Distance);
- whereas in this case KEN is between RICK and EARL, with whom he shares the High School and Burglar characteristics;
- the maximum number of links is 3 and the greatest power of clustering belongs to MIKE;
- NICK, RICK and DAVE have the highest protection grade (7).

Figure 3. MST graph resulting from the Prior Probability distances matrix.

**Back Propagation**

In this case the clustering is 87.5% correct.

- in fact, DON (Sharks) is linked to AL (Jets) while DAVE and RICK (Sharks) depend on GREG (Jets), and they are very far from the other Sharks;
- in this case, the boundary line is between MIKE and IKE that, like DOUG and NEAL, have the same characteristics (excluding the gang);
- the maximum number of links with AL is 4, while the greatest power of clustering belongs to GREG and KEN;
- AL and MIKE have the highest protection grade.


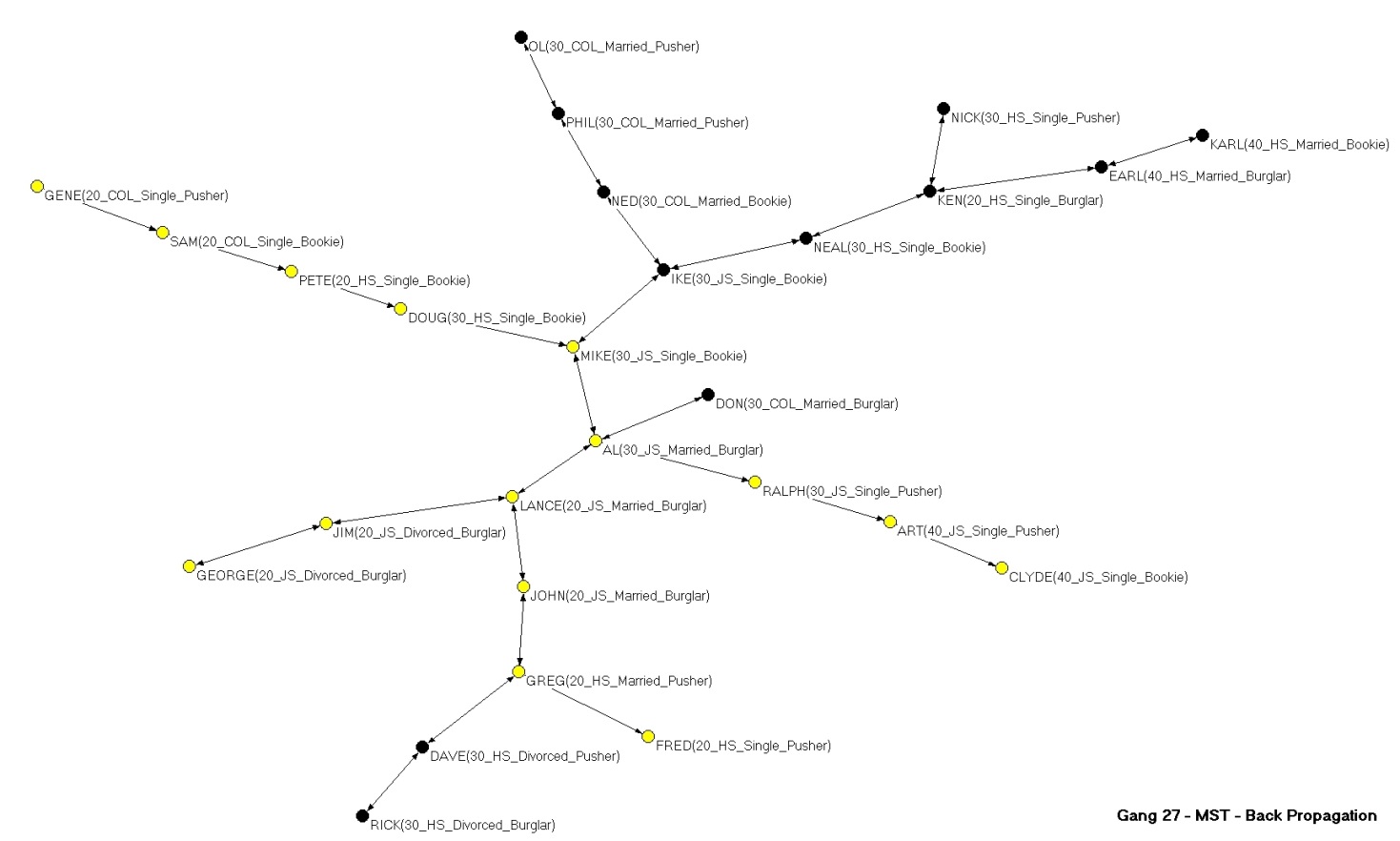


Figure 4. MST graph resulting from Back Propagation distances matrix.

**Auto Contractive Map**

In this case we can see very precise clustering (100%) and distribution (fig. 5). The most interesting observations are as follows:

- the separation between the two clusters coincides with two subjects (MIKE and IKE), who have the same characteristics but belong to rival gangs;
- the Jets cluster is split into 4 ramifications linked to MIKE, hence more complex than the Sharks’, which is more linear;
- GREG is the only subject who appears to be unrelated to the rest of the Jets cluster; in fact, he is the only one to be Married with High School and quite rightly he is linked to FRED, who is the only Pusher with High School in the Jets and, together, have 3 characteristics in common;
- where all the Jets subjects have the Burglar characteristic (AL, LANCE, JOHN, GEORGE and JIM) the tree section has a different and more coherent logic than the one shown in the other graphs, for example the Prior Probability graph; in fact, LANCE forms a hub with 3 links;
- the two branches where the Sharks subjects also have the Married characteristic are organised in a way that reflects the subjects’ characteristics: on one branch we have EARL and KARL, who are the only 40-year-old ones, on the other branch we have DON, OL, NED and PHIL, all aged 30, and COL;
- moreover, the CM is very precise in positioning closer to the Jets those Sharks with the Single characteristic, which is typical of the Jets;
- the maximum number of links with MIKE is 5; he also has the greatest power of clustering;
- NEAL and IKE have the highest protection grade.


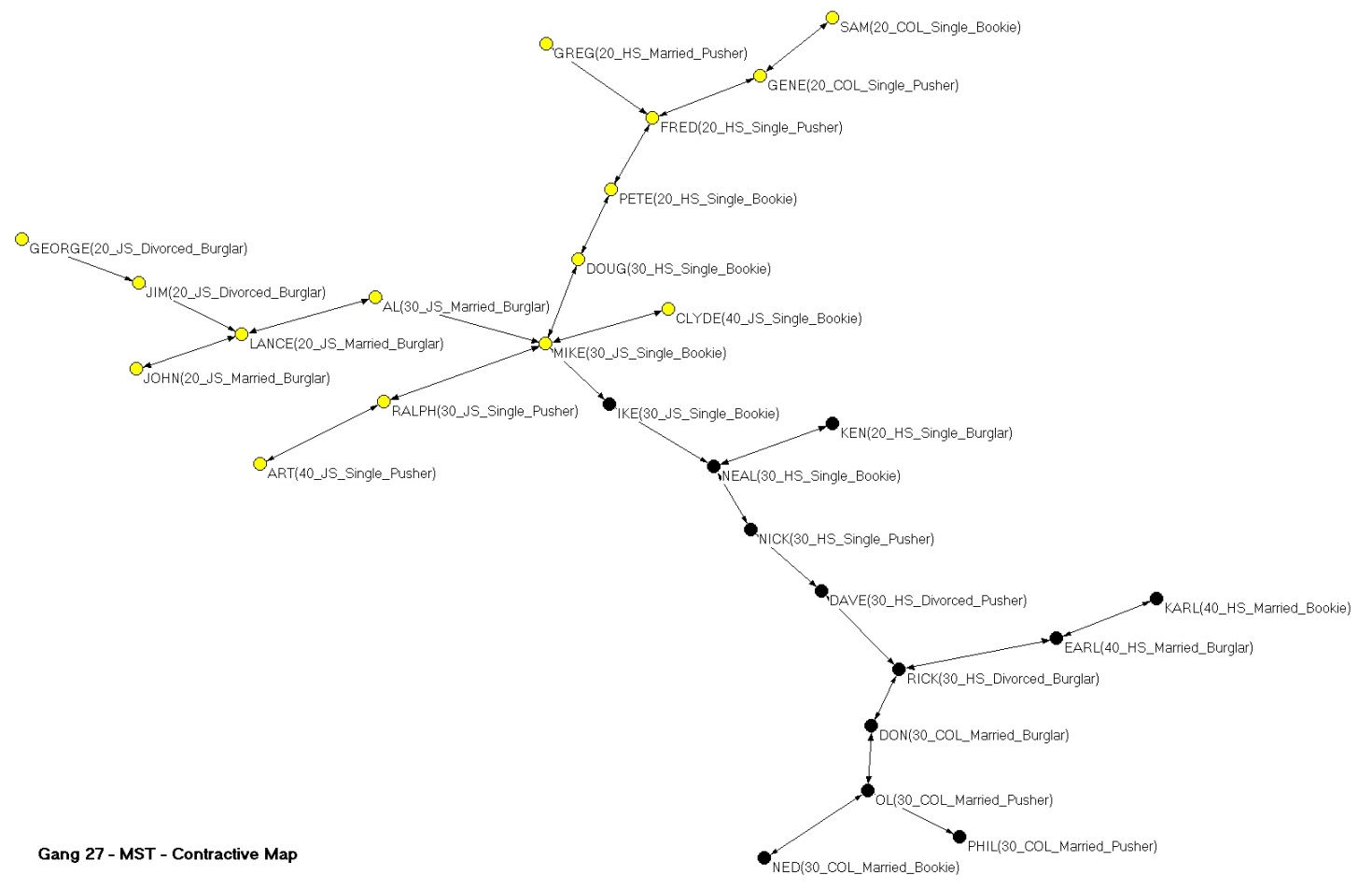


Figure 5. MST graph resulting from Contractive Map distances matrix.

|  | **Num** | **Euclidean Distance** | **% Corr** | **Errors** | **Names** |
| --- | --- | --- | --- | --- | --- |
| Jets | 15 | 15 | 100.0% | 0 |  |
| Sharks | 12 | 10 | 83.3% | 2 | IKE, KEN |
|  |  |  |  |  |  |
|  | **Num** | **Linear Correlation** | **% Corr** | **Errors** | **Names** |
| Jets | 15 | 15 | 100.0% | 0 |  |
| Sharks | 12 | 11 | 91.7% | 1 | KEN |
|  |  |  |  |  |  |
|  | **Num** | **Prior probability** | **% Corr** | **Errors** | **Names** |
| Jets | 15 | 10 | 66.7% | 5 | AL, JOHN, LANCE, GEORGE |
| Sharks | 12 | 11 | 91.7% | 1 | IKE |
|  |  |  |  |  |  |
|  | **Num** | **Back propagation** | **% Corr** | **Errors** | **Names** |
| Jets | 15 | 15 | 100.0% | 0 |  |
| Sharks | 12 | 9 | 75.0% | 3 | DON, DAVE, RICK |
|  |  |  |  |  |  |
|  | **Num** | **Contractive Map** | **% Corr** | **Errors** | **Names** |
| Jets | 15 | 15 | 100.0% | 0 |  |
| Sharks | 12 | 12 | 100.0% | 0 |  |

Details and equations for the other algorithms can be found in:

(1) Massimo Buscema (ed), Auto Contractive Maps, Semeion Techinal Paper 32, Aracne Publisher 2008, Rome, ISBN 978–88–548–1714–2;

(2) Massimo Buscema and Pier L. Sacco, *Auto-contractive Maps, the H Function, and the Maximally Regular Graph (MRG): A New Methodology for Data Mining,* in V. Capecchi et al. (eds.), Applications of Mathematics in Models, Artificial Neural Networks and Arts, Chapter 11, DOI 10.1007/978-90-481-8581-8_11, Springer Science+Business Media B.V. 2010).
