# Supplementary material for: Networks in Coronary Heart Disease Genetics As a Step towards Systems Epidemiology
Source: PLoS One. 2015 May 7;10(5):e0125876. doi: 10.1371/journal.pone.0125876 (PMC4423836; doi:10.1371/journal.pone.0125876)
Supplement: S4 Appendix — (DOCX) [file pone.0125876.s004.docx]

**Appendix 4**

**The Maximally Regular Graph (MRG)**

**By Massimo Buscema**

The minimum spanning tree (MST) described earlier represents what we could call the ‘nervous system’ of any dataset. In fact, summing up all of the connection strengths among all the variables, we get the total energy of that system. The MST selects only the connections **that minimize this energy**, i.e., the only ones that are really necessary to keep the system coherent. Consequently, all the links included in the MST are fundamental, but, on the contrary, not every ‘fundamental’ link of the dataset need be in the MST. Such limit is intrinsic to the nature of MST itself: every link that gives rise to a cycle into the graph is eliminated, whatever its strength and meaning. To fix this shortcoming and to better capture the intrinsic complexity of a dataset, it is necessary to add more links to the MST, according to two criteria:

- the new links have to be **relevant** from a quantitative point of view;
- the new links have to be able to generate new **cyclic regular microstructures**, from a qualitative point of view.

Consequently, the MST tree-graph is transformed into an undirected graph with cycles. Because of the cycles, the new graph is a dynamic system. Due to this, the new graph should provide information not only about the structure, but also about the **functions** of the variables of the dataset. The new graph can be built as follows:

- assume the MST structure as the starting point of the new graph;
- consider the list of the connections skipped during the derivation of the MST;
- estimate the H Function of the new graph each time that you add a new connection to the MST basic structure and monitor the variation of the complexity of the new graph after each step.

We will call **Maximally Regular Graph** (MRG) the graph with the highest **H Function** value among all the graphs generated by adding back to the original MST, one by one, the missing connections previously skipped during the computation of the MST itself.

The process can be described in a mathematical way as follows:

The global hubness index H_0_ of a connected graph of N nodes can be calculated as

1. $H_{0}=\frac{\mu\cdot\phi- 1}{A}$;

where *Α* is the number of links in the graph; $\mu=\frac{A}{M}$ where Μ is the number of iterations of the pruning algorithm; and $\varphi=\frac{1}{P}\sum_{J}^{P} S_{TG j}$ where P is the number of types of pruning and *S_TG j_* is the series of pruning gradient types at the j-th iteration.

The generic function on a graph with *A_p_* arcs and *N* nodes is:

(2) $H_{i}=f(G(A_{p},N))$

where *G* is the gradient of the erased nodes at cycle *j*.

Then the calculation of *H* Function, where *H*_0_ represents MST complexity is:

$$H_{i}=\frac{\mu_{p}\cdot\phi_{p} - 1}{A_{p}}$$

and the MRG graph can be defined as:

$$MRG=Max\{H_{i}\}$$

with $i\in[0,1,2,...,R]$ the index of the H function

$p\in[N-1,N,N+1,...,N-1+R]$ the index for the number of graph arcs

and $R\in\left[0,1,..,\frac{(N-1)\cdot(N-2)}{2}\right]$ the number of the skipped arcs during the MST generation.

*R* is a key variable during the computation of the MRG and can be null, when the computation of the MST calls for no connections to be skipped. In this case, there is no MRG for that dataset. *R* also makes sure that the last - and consequently the weakest - connection added to generate the MRG is more relevant that the weakest connection of the MST. The MRG, starting from the MST, generates the graph presenting the **highest number of regular microstructures that make use of the most important connections** of the dataset. The higher the value of the H Function at the selected connections to generate the MRG, the more meaningful the microstructures of the MRG.
